# Fungal F8-Culture Filtrate Induces Tomato Resistance against Tomato Yellow Leaf Curl Thailand Virus

**DOI:** 10.3390/v13081434

**Published:** 2021-07-23

**Authors:** Yi-Shu Chiu, Yuh Tzean, Yi-Hui Chen, Chi-Wei Tsai, Hsin-Hung Yeh

**Affiliations:** 1 Agricultural Biotechnology Research Center, Academia Sinica, Taipei 11529, Taiwan; abookchiu@gmail.com (Y.-S.C.); miketzean@gmail.com (Y.T.); yihui820123@gate.sinica.edu.tw (Y.-H.C.); 2 Department of Entomology, National Taiwan University, Taipei 10617, Taiwan; chiwei@ntu.edu.tw; 3 Department of Plant Pathology and Microbiology, National Taiwan University, Taipei 10617, Taiwan; 4 Institute of Biotechnology, National Taiwan University, Taipei 10617, Taiwan

**Keywords:** tomato yellow leaf curl disease, whitefly transmitted begomoviruses, tomato yellow leaf curl Thailand virus, virus resistance, plant immunity inducer

## Abstract

Tomato (*Solanum lycopersicum*) is an important economic crop worldwide. However, tomato production is jeopardized by the devastating tomato yellow leaf curl disease caused by whitefly-transmitted begomoviruses (WTBs). In this study, we evaluated the efficacy of our previously developed plant antiviral immunity inducer, fungal F8-culture filtrate, on tomato to combat tomato yellow leaf curl Thailand virus (TYLCTHV), the predominant WTB in Taiwan. Our results indicated that F8-culture filtrate treatment induced strong resistance, did not reduce the growth of tomato, and induced prominent resistance against TYLCTHV both in the greenhouse and in the field. Among TYLCTHV-inoculated Yu-Nu tomato grown in the greenhouse, a greater percentage of plants treated with F8-culture filtrate (43–100%) were healthy-looking compared to the H_2_O control (0–14%). We found that TYLCTHV cannot move systemically only on the F8-culture filtrate pretreated healthy-looking plants. Tracking the expression of phytohormone-mediated immune maker genes revealed that F8-culture filtrate mainly induced salicylic acid-mediated plant immunity. Furthermore, callose depositions and the expression of the pathogen-induced callose synthase gene, *POWDERY MILDEW RESISTANT 4* were only strongly induced by TYLCTHV on tomato pretreated with F8-culture filtrate. This study provides an effective way to induce tomato resistance against TYLCTHV.

## 1. Introduction

Tomato (*Solanum lycopersicum*) is an economically important vegetable crop, which ranks third in this class in terms of volume of production and first in terms of processing volume [1]. However, tomato yellow leaf curl disease (TYLCD) caused by whitefly-transmitted begomoviruses (WTBs) has a devastating effect on tomato production worldwide [2,3,4,5]. One of the main approaches to managing TYLCD relies on controlling the whitefly vector using insecticide [6,7]. However, it has become increasingly difficult to control the whitefly vector population in this way because of the rapid evolution of whitefly’s resistance to insecticides [2,7,8].

Screening of TYLCD-resistant tomato cultivars revealed that most commercially cultivated tomato cultivars are susceptible to the disease [9]. To breed TYLCD-resistant cultivars, six TYLCD resistance gene loci (Ty gene family) were identified from wild tomato species [6,10,11,12]. However, the breeding process requires several backcross steps and is usually time consuming. As a large number of tomato cultivars are planted for commercial purposes, transferring the resistance loci to these various commercial tomato cultivars remains a challenge. Transgenic approaches that mainly use RNAi strategies have been successfully applied to plants to manage TYLCD infection with promising results [13,14,15,16,17,18,19,20,21,22,23,24]. However, the debate over using genetically modified organisms (GMO) in agriculture has hindered the distribution of these transgenic tomatoes. One way to address this issue would be to induce plant innate immunity to combat TYLCD.

Plant innate immunity consists of two distinctive pathways: microbe/pathogen-associated molecular pattern (MAMP/PAMP)-triggered immunity (PTI) and effector-triggered immunity (ETI) [25,26,27,28,29]. PTI is triggered by MAMPs/PAMPs via cell surface-localized pattern-recognition receptors (PRRs), whereas ETI is activated by pathogen effector proteins predominantly through direct or indirect perception of intracellular nucleotide-binding, leucine-rich repeat receptors (NLRs) [25,30,31,32]. At the first layer of plant innate immunity, PTI, signals are transmitted through activation of mitogen-activated protein kinases (MAPKs), inducing signaling and defense processes such as reactive oxygen species (ROS) production, ion fluxes, salicylic acid (SA) and ethylene (ET) accumulation, resistance gene activation such as genes encoding pathogenesis-related proteins (PRs), and callose deposition [33,34,35,36,37,38]. However, adapted pathogens can deliver diverse effectors into plant cells to interfere with PTI and enhance virulence [39,40,41,42,43]. To counteract the invasion, plants utilize intracellular polymorphic resistance (R) proteins to perceive these effectors either through direct binding or monitoring perturbations of host proteins caused by effectors, to initiate a more robust defense ETI [29,44]. ETI often leads to hypersensitive response (HR), a type of programmed cell death that has been hypothesized to restrict pathogen growth at the local infection site [32,45,46,47].

The application of ETI in plant protection requires introduction of R protein into susceptible cultivars by breeding or transgenic approaches, which is time consuming, as mentioned above. In addition, WTBs evolve quickly which leads to the occurrence of resistance breaking strains as R protein usually recognizes specific effectors. In comparison to ETI, PTI can be induced in all cultivars, and has the potential to be applied to all tomato cultivars. Indeed, several plant immunity inducers from biological sources have been reported including proteins [48,49,50,51,52,53], oligosaccharides [54,55], microbial inducers [56,57,58], and oil and related compounds [59]. However, the application of plant immunity inducers on crop protection requires the need to overcome several challenges, such as the high cost of the inducers, effectiveness in plant protection, and reduced crop growth and quality [60,61]. Therefore, practical application of plant immunity inducers on crops for effective protection mostly remains to be explored.

In our previous study, we screened culture filtrates derived from soil microorganisms cultured with medium made of weeds and cheaply obtainable vegetables (may contain pathogen/damage-associated molecular patterns) for induction of effective plant defense against viruses [62]. We obtained a culture filtrate derived from *Trichosporon* sp. (F8-culture filtrate) that induced strong resistance to different viruses in various plants [62]. The infection rate of tobacco mosaic virus (TMV) reduced dramatically on *Nicotiana benthamiana* pretreated with F8-culture filtrate [62]. The symptoms were also considerably reduced on F8-culture filtrate-pretreated *N. benthamiana* and *Brassica juncea* inoculated with cucumber mosaic virus (CMV) and turnip mosaic virus (TuMV), respectively. A fraction of the polysaccharides, designated F8-polysaccharide, with estimated molecular weight of 4260–12,000 kDa is responsible for induction of the plant antiviral immunity. The F8-polysaccharide mainly induced priming of the SA immune pathway, and therefore, has great potential for application on tomato against viruses as previous reports have also indicated that SA-mediated plant immunity is important in tomato resistance to viral diseases including TYLCD [63]. 

In this study, we applied F8-culture filtrate to tomato to combat tomato yellow leaf curl Thailand virus (TYLCTHV), the predominant WTB in Taiwan [64,65,66]. Our results indicated that F8-culture filtrate treatment in tomatoes induced strong resistance to TYLCTHV both in the greenhouse and in the field. Furthermore, we resolved the main immune responses in F8-culture filtrate-treated tomato. Our results not only provide an effective method to manage TYLCD, but also provide important knowledge for further design strategies to combat the effect of WTBs on crops.

## 2. Materials and Methods

### 2.1. Whiteflies, Virus and Plants

The sources of our whitefly (*Bemisia tabaci* Middle East–Asia Minor 1, MEAM1) and TYLCTHV isolate Ly5 (GenBank Accession No. GU723742 and GU723754) are described in Weng et al. [67].

The non-viruliferous whitefly was reared on Chinese kale (*Brassica oleracea* cv. Alboglabra Group) in whitefly-proof net cages (30 × 30 × 30 cm^3^) at 26 °C, under a 16-h light/8-h dark cycle.

TYLCTHV isolate Ly5 was maintained, as previously described [68], in tomato plants (*Solanum lycopersicum* cv. ANT22) and used for vector transmission, using *B*. *tabaci*, under the aforementioned conditions. 

Tomato cultivar Yu-Nu (*S. lycopersicum*) was purchased from Known-You Seed (Kaohsiung, Taiwan), and grown and maintained in a glass-covered greenhouse. Xiao-Nu (*S*. *lycopersicum*) was purchased from Known-You Seed and maintained in the field (Alian District, Kaohsiung City, Taiwan) for field analysis.

### 2.2. Treatment and Inoculation

Yu-Nu tomatoes (2–3 weeks after sowing) were sprayed with H_2_O (0.4 mL per plant) or F8-culture filtrate (0.4 mL per plant) for a total of three times with a 24-h interval between each spray as previously described [62]. Twenty-four hours after the final treatment, plants were inoculated with TYLCTHV by viruliferous *B. tabaci* as previously described [69] with modifications. Briefly, non-viruliferous *B. tabaci* was transferred to the net cage enclosed with TYLCTHV-infected ANT22 tomato and allowed 3-day acquisition access periods (AAP). The viruliferous *B*. *tabaci* was used to inoculate tomato plants through feeding for 96 h (approximately 20 whiteflies on one leaf per plant). The inoculated-leaves were cut and collected at 4 dpi. The number of replicated experimental sets and the number of biological replicates for each assay are specified in the Results section.

### 2.3. Disease Index for Evaluation of Tomato Yellow Leaf Curl Disease (TYLCD)

Tomatoes were evaluated for TYLCD severity according to a disease index of 5 levels. Level 0 = no visible symptoms, inoculated plants show the same growth and development as non-inoculated plants; Level 1 = slight yellowing and minor curling of leaflet ends; Llevel 2 = wide range of leaf yellowing, curling and cupping, with some reduction in size; Level 3 = wide range of leaf yellowing, pronounced leaf cupping and curling; Level 4 = very severe plant stunting and leaf yellowing, pronounced leaf cupping and curling, and plant ceased to grow.

### 2.4. Nucleic Acid Isolation and Gene Expression Analysis

Total DNA and RNA were extracted using an optimized CTAB method [70]. Leaf tissue (100 mg) was frozen with liquid nitrogen and ground into a fine powder with a pestle and mortar. The powder was then dissolved in 750 μL of CTAB buffer (2% CTAB, 2% PVP, 2.0 M NaCl, 0.1 M Tris–HCl, 25 mM EDTA, 4% β-mercaptoethanol) and incubated at 65 °C for 30 min. Next, 750 μL of chloroform:IAA (24:1) was added to the mixture and centrifuged at 16,000× *g* for 10 min. To precipitate DNA and RNA, the upper phase was transferred to a new tube, mixed with 2 volumes of ethanol (99.9% *v*/*v*) and 1/4 volume of 10 M LiCl, and centrifuged at 16,000× *g* for 10 min. The DNA and RNA supernatant were discarded and the pellet was washed with 1 mL of ethanol (70% *v*/*v*). After centrifugation, the supernatant was discarded and the pellet was dissolved in 200 μL nuclease-free water (Invitrogen, Carlsbad, CA, USA). 

Residual DNA was removed by using the TURBO DNA-free kit (Ambion, Austin, TX, USA) before RT-PCR. cDNA was synthesized by using the PrimeScript RT-PCR Kit (Takara Bio, Shiga, Japan). Quantitative PCR (q-PCR) and quantitative reverse transcription PCR (qRT-PCR) were conducted with the SYBR protocol (Life Technologies, Carlsbad, CA, USA) and the ABI 7100 real-time PCR system (Applied Biosystems, Carlsbad, CA, USA). For quantitative analysis, the common tomato endogenous control, β-actin [71,72,73], was verified for expression stability among different samples and the primer efficiency compared to specified target gene(s) was conducted prior to use. Relative fold change in DNA expression was determined by calculating 2^−ΔΔCt^. The sequences of primers used for analysis are listed in Table S1.

### 2.5. Measurement of Tomato Plant Fresh Weight

Tomato Yu-Nu (3 weeks after sowing) were pretreated with H_2_O or F8-culture filtrate as mentioned above. All shoot tissues of individual plants were measured for fresh weight. 

### 2.6. Staining of Callose Depositions

The tomato plants were pretreated with either H_2_O or F8-culture filtrate. Leaf samples were collected 24 h post treatment. One leaf per plant (*n* = 7) with similar growth stage were collected for each treatment group. In addition, for inoculation, viruliferous *B. tabaci* was used to inoculate on 1 leaf per tomato plant. One inoculated leaf per plant (*n* = 7) was also collected at 4 days post inoculation for staining of callose deposits. The collected leaves were fixated and de-stained with 1:3 of acetic acid (100%)/ethanol (100%) overnight. After washing in 150 mM K_2_HPO_4_ for 30 min, leaves were incubated for 2 h in staining solution (150 mM K_2_HPO_4_ and 0.01% aniline blue). Zeiss Imager Z1 fluorescence microscope (Carl Zeiss AG, Oberkochen, Germany) using a DAPI filter was used for imaging. The microscopic field photos were printed and the number of callose depositions were counted manually. 

### 2.7. Field Trial

The field trials were conducted in Yuchi Township, Nantou County, Taiwan, and Alian District, Kaohsiung City, Taiwan. Three-week-old (after sowing) Yu-Nu and Xiao-Nu tomato cultivar seedlings were sprayed with H_2_O or F8-culture filtrate 3 times with a 24 h interval between each spray. Twenty-four hours after the final treatment, plants were transferred to an open field located at the specified location. The tomato plants were evaluated for disease severity at 2 months after transplantation. Evaluation of disease severity was conducted manually and recorded for each tomato plant based on 5 levels in disease index (see 2.3 Disease Index for Evaluation of Tomato Yellow Leaf Curl Disease and Figure S1).

## 3. Results

### 3.1. F8-Culture Filtrate Induced Virus Resistance in Tomato Cultivar Yu-Nu

**Figure 1:**
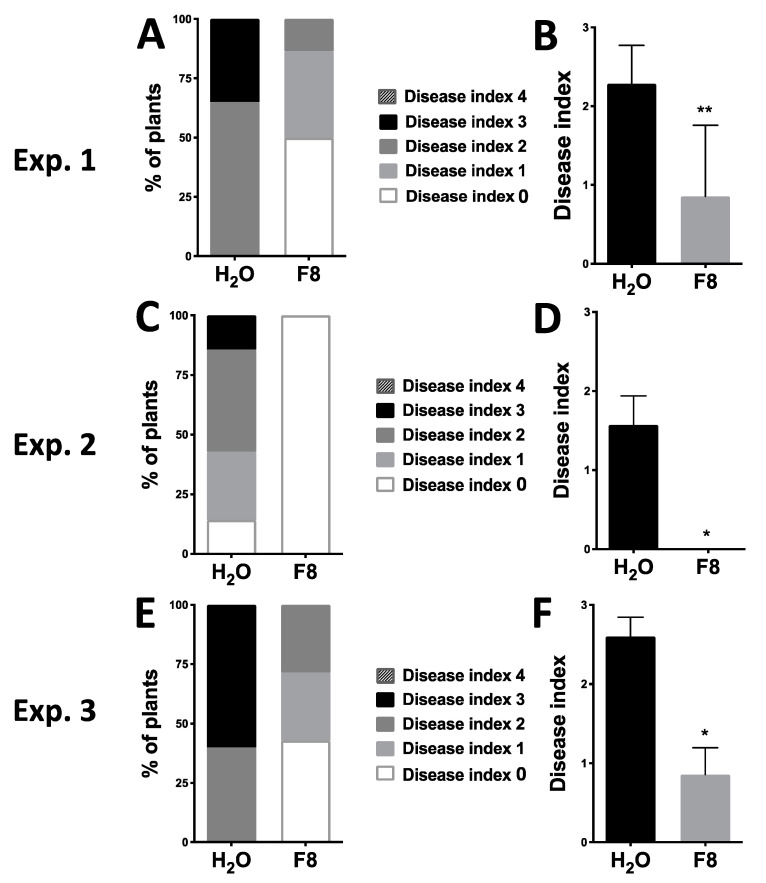
Symptoms on H_2_O or F8-culture filtrate pretreated Yu-Nu tomato plants inoculated with tomato yellow leaf curl Thailand virus (TYLCTHV). The level of disease index was recorded at 28 days post inoculation (dpi) in the H_2_O or F8-culture filtrate pretreated group (**A**,**C**,**E**). The average disease index of plants inoculated with TYLCTHV according to the specified pretreatment groups (**B**,**D**,**F**). Data represent mean ±  SEM; *n* = 6–7 biological replicates. Student’s *t*-test was used for comparative analysis of the data for the F8-culture filtrate pretreated group and the H_2_O pretreated control group. * *p* < 0.05; ** *p* < 0.01.

To analyze whether F8-culture filtrate can induce tomato resistance against WTBs, we selected the cherry tomato cultivar, Yu-Nu, for analysis. Yu-Nu tomato is a favorable tomato with commercial value; however, it is known for its susceptibility to several pathogens including WTBs. Initially, we conducted our experiments in a greenhouse. We used the whitefly vector, *Bemisia tabaci*, as a vector to transmit the TYLCTHV from infected tomato to Yu-Nu. Yu-Nu tomatoes were pretreated with H_2_O or F8-culture filtrate by spraying consecutively three times with a 24 h interval between each spray. All pretreated plants were inoculated with TYLCTHV by using whitefly vector at 24 h post treatment (hpt). The disease severity was scored using an established disease index (Figure S1) on all TYLCTHV-inoculated plants at 28 days post inoculation (dpi). In our 3 repeated sets of experiments (Exp. 1, 2, and 3), we analyzed 6–7 plants per treatment group (H_2_O or F8-culture filtrate pretreatment) in each of the experimental sets (Figure 1). Most H_2_O pretreated plants showed more severe symptoms than the F8-culture filtrate pretreated group (Figure 1). Healthy-looking (Disease Index 0) plants were observed more in the F8-culture filtrate pretreated group compared to the H_2_O pretreated plants (Figure 1A,C,E and Figure S2).

**Figure 2:**
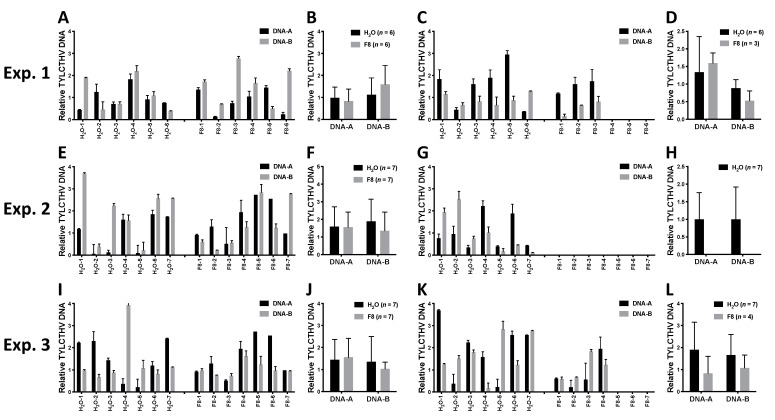
Quantification of TYLCTHV genomic DNA in inoculated tomato plants pretreated with F8-culture filtrate or H_2_O. Relative TYLCTHV DNA level was quantified from H_2_O or F8-culture filtrate pretreated plants corresponding to Exp. 1, 2, and 3 in Figure 1 by qPCR analysis. TYLCTHV DNA was detected from individual samples of TYLCTHV-inoculated leaves (**A**,**E**,**I**) or systemic leaves (**C**,**G**,**K**). Three technical replicates were performed for all samples. Data represent mean ±  SEM. β-actin was used as an input control. The average of TYLCTHV DNA detected from (**A**,**E**,**J**) are shown in (**B**,**F**,**J**), respectively. The average of TYLCTHV DNA detected from (**C**,**G**,**K**) are shown in (**D**,**H**,**L**), respectively. Data represent mean ±  SEM; *n* = 3–6 biological replicates. β-actin was used as an input control. Student’s *t*-test was used for comparative analysis of the data for the F8-culture filtrate pretreated group and the H_2_O pretreated control group.

From the same 3 sets of experiments (Exp. 1, 2, and 3), the inoculated leaf and one systemic leaf from each plant (*n* = 6–7) were collected for evaluation of TYLCTHV accumulation in the H_2_O or F8-culture filtrate pretreated group. The relative accumulation of TYLCTHV genomic DNAs, DNA-A and -B, were quantified by quantitative PCR (qPCR). The results showed that TYLCTHV DNAs could be detected in inoculated leaves on plants pretreated with H_2_O or F8-culture filtrate (Figure 2A,B,E,F,I,J). On systemic leaves, TYLCTHV DNAs can be detected for both H_2_O or F8-culture filtrate pretreated plants that showed disease symptoms (Figure 1A,C,E and Figure 2C,D,G,H,K,L). However, TYLCTHV could not be detected on F8-culture filtrate pretreated healthy-looking plants (Figure 2C,G,K). Of note, even though we could observe healthy-looking plants in the H_2_O pretreated group in Exp. 2 (Figure 1C), we could still detect TYLCTHV on the systemic leaves of these plants (Figure 2G). The experiments were repeated for 3 times with similar results. 

### 3.2. Mock and F8-Culture Filtrate Treated Plants Show Similar Fresh Weight

**Figure 3:**
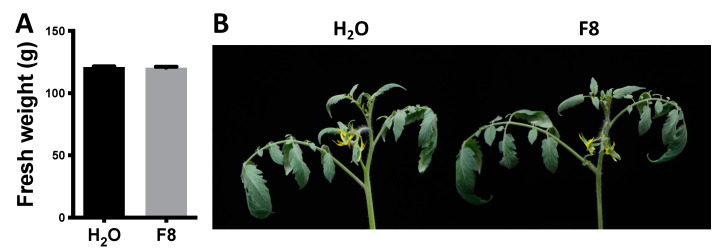
Fresh weight of Yu-Nu tomato with H_2_O or F8-culture filtrate pretreatment. (**A**) The average fresh weight was recorded for each treatment group (*n* = 11). (**B**) The phenotype of H_2_O or F8-culture filtrate at 5 weeks post treatment.

To determine whether F8-culture filtrate treatment affects the fresh weight in tomato, 3-week-old Yu-Nu were pretreated with H_2_O or F8-culture filtrate as mentioned above and analyzed the fresh weight of the tomato plants 5 weeks post treatment. We conducted 2 sets of experiments and analyzed 8–11 plants per treatment (H_2_O or F8-culture filtrate) in each of the experimental sets. We found that H_2_O and F8-culture filtrate-treated tomato showed similar fresh weight in our 2 replicated sets of experiments (Figure 3 and Figure S3).

### 3.3. F8-Culture Filtrate Mainly Induced the Expression of SA-Responsive Immune Marker Genes on Tomato Cultivar Yu-Nu

**Figure 4:**
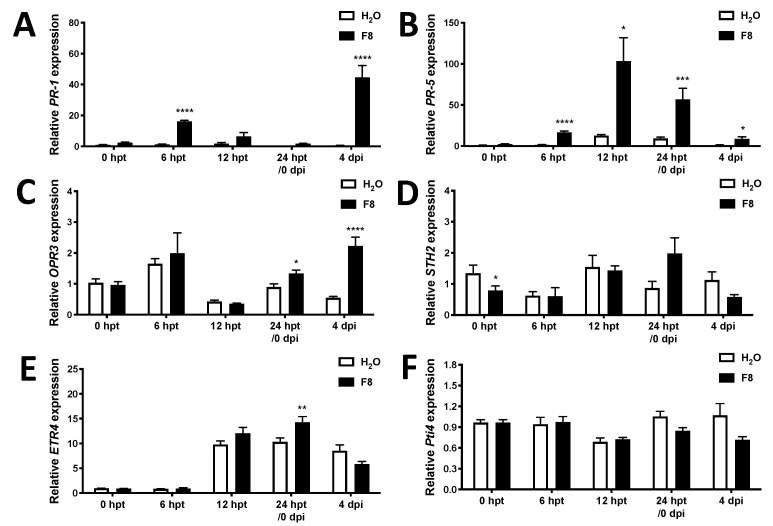
The expression of phytohormone-mediated immune marker genes in F8-culture filtrate or H_2_O pretreated Yu-Nu tomato before or after TYLCTHV inoculation. Tomato plants were pretreated with H_2_O or F8-culture filtrate and inoculated with TYLCTHV using whitefly vector at 24 hpt. The relative expression of SA-responsive gene *PR-1* (**A**) and *PR-5* (**B**), jasmonic acid (JA)-biosynthesis gene *OPR3* (**C**), JA-responsive gene *STH2* (**D**), and ethylene (ET)-responsive gene *ETR4* (**E**) and *Pti4* (**F**) were detected by qRT-PCR at 0, 12 and 24 hpt or at 4 dpi. β-actin was used as an input control. Data are mean ± SEM of 3 biological replicates. Student’s *t*-test was used for comparative analysis of the data for the H_2_O and F8-culture filtrate pretreated groups. * *p* < 0.05; ** *p* < 0.01, *** *p* < 0.005, **** *p* < 0.001.

To understand the possible immune pathways involved in F8-culture filtrate-induced resistance against TYLCTHV, we detected the expression of phytohormone-responsive immune marker genes. A comparison of the F8-culture filtrate and H_2_O pretreated Yu-Nu tomato showed that the expression of SA-responsive immune marker gene, *PR-1*, increased 12.4-fold at 6 hpt, and was robustly induced by TYLCTHV to 82.8-fold at 4 dpi (Figure 4A). In addition, the expression of *PR-5* increased 12.2-fold at 6 hpt, robustly increased 8.1-fold at 12 hpt, with the induction gradually decreasing to 6.1-fold at 24 hpt. Of note, after TYLCTHV infection, the *PR-5* expression remained higher in F8-culture filtrate than H_2_O pretreated Yu-Nu tomatoes, but the induction fold was not as prominent as at 6, 12 and 24 hpt (Figure 4B).

In F8-culture filtrate-pretreated Yu-Nu tomato, we found that the expression of JA-responsive immune marker gene, *OPDA reductase 3* (*OPR3*) [74], was increased at 24 hpt (1.5 fold) and 4 dpi (4.1 fold) (Figure 4C) compared to H_2_O pretreated Yu-Nu tomato, but the induction of another JA-responsive immune marker gene, *STH2* [75], was not observed in H_2_O or F8-culture filtrate-pretreated Yu-Nu with or without TYLCTHV infection (Figure 4D).

Two ethylene (ET)-responsive immune marker genes, *ethylene receptors-4* (*ETR4*) [76] and tomato transcription factor *Pti4* [77], were also selected for analysis. The results showed that *ETR4* only increased in F8-culture filtrate-pretreated Yu-Nu as compared to the H_2_O pretreated Yu-Nu control at 24 dpi (1.4 Fold) (Figure 4E), and the induction of *Pti4* was not observed in H_2_O or F8-culture filtrate pretreated Yu-Nu with or without TYLCTHV infection (Figure 4F).

### 3.4. F8-Culture Filtrate Induced Callose Deposition on TYLCTHV-Inoculated Tomato Cultivar Yu-Nu

**Figure 5:**
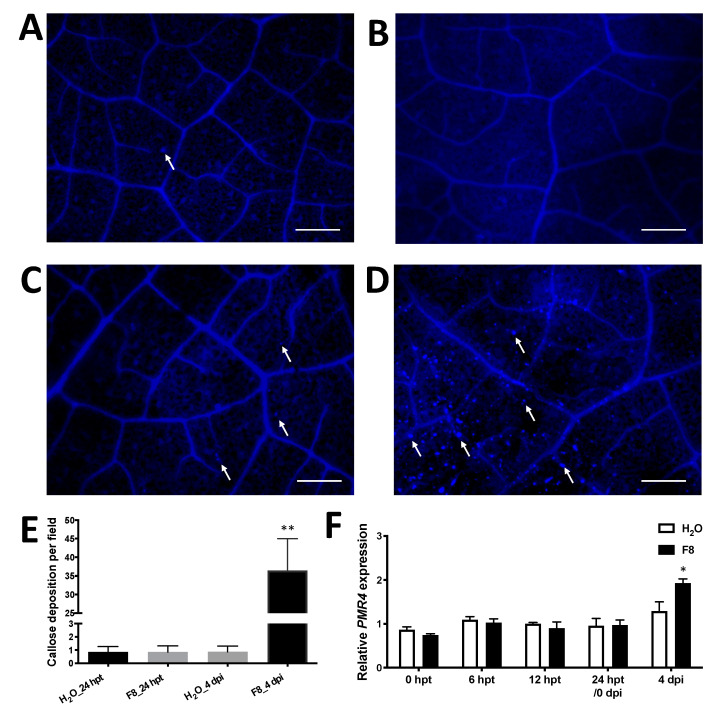
Staining of callose deposition in F8-culture filtrate or H_2_O pretreated Yu-Nu tomato before or after TYLCTHV inoculation. Tomato plants were pretreated with H_2_O (**A**,**C**) or F8-culture filtrate (**B**,**D**). The pretreated plants were inoculated with TYLCTHV using whitefly vector at 24 hpt. The callose deposition was stained with 0.02% aniline blue. Callose depositions were analyzed at 24 hpt without TYLCTHV infection (**A**,**B**) or with TYLCTHV infection at 4 dpi (**C**,**D**) using a Zeiss Imager Z1 fluorescence microscope. Scale bar = 200 μm. White arrow indicates callose depositions. (**E**) The average numbers of callose deposition per field. Two pictures were taken per leaf (TYLCTHV-inoculated) collected from each plant (*n* = 7 plants) from H_2_O or F8-culture filtrate treatment for analysis. Data shows mean number of callose deposition per field ± SEM. Student’s *t*-test was used to analyze the data from the F8-culture filtrate pretreated group and the H_2_O-pretreated control group. ** *p* < 0.01. (**F**) The relative expression of callose synthase gene *PMR4* was detected by qRT-PCR at 0, 12 and 24 hpt without TYLCTHV infection or with TYLCTHV infection at 4 dpi. Data are mean ± SEM from 3 plants. β-actin was used as an input control. Student’s *t*-test was used for comparative analysis of the data in the H_2_O and F8-culture filtrate pretreated groups. * *p* < 0.05.

To understand how plants pretreated with F8-culture filtrate confine TYLCTHV infection (Figure 2), we analyzed whether callose deposition occurred in the tomato plants. Leaves were randomly collected from H_2_O or F8-culture filtrate pretreated plants at 24 hpt and stained with 0.2% aniline blue (Figure 5A,B). In addition, we also collected leaves from plants pretreated with H_2_O or F8-culture filtrate with TYLCTHV inoculation at 4 dpi for staining (Figure 5C,D). Fluorescence microscopy was used to examine the callose deposition under UV illumination. All images were used for quantification of callose deposition. We observed limited callose deposition on H_2_O or F8-culture filtrate pretreated plants at 24 hpt (Figure 5A,B,E). On TYLCTHV inoculated leaves, a limited number of callose depositions were observed on leaves pretreated with H_2_O (Figure 5C,E), but more profound callose depositions were observed only on leaves of plants pretreated with F8-culture filtrate (Figure 5D,E).

In addition to the staining of callose deposition, we also analyzed the expression of the pathogen-induced callose synthase gene, *POWDERY MILDEW RESISTANT 4* (*PMR4*) [78]. Nucleic acids were extracted from collected leaves as mentioned above for callose staining. Significant induction of *PMR4* was only observed on leaves of plants pretreated with F8-culture filtrate following TYLCTHV inoculation at 4 dpi (Figure 5F). The expression of *PMR4* is consistent with the staining of callose deposition. 

### 3.5. F8-Culture Filtrate Induced Virus Resistance on Tomato Cultivar Yu-Nu and Xiao-Nu in the Field

**Figure 6:**
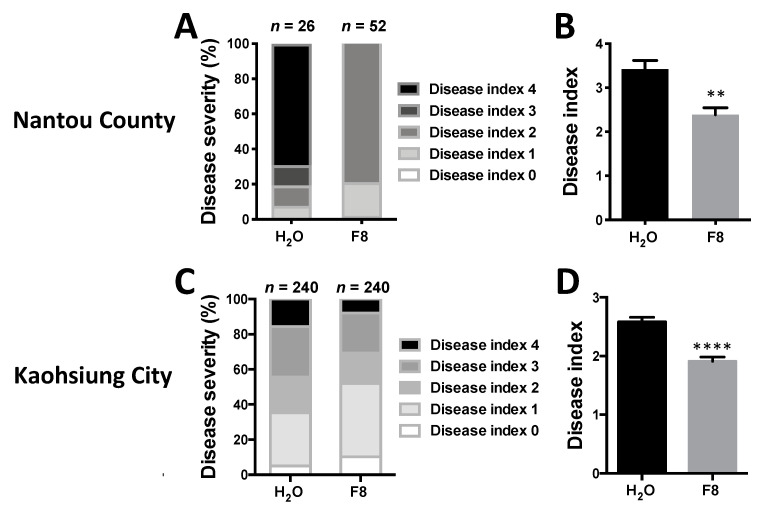
Symptoms on H_2_O or F8-culture filtrate treated tomato in the field in Nantou County and Kaohsiung City. Tomato seeds were sown and grown in a growth chamber for 3 weeks. Yu-Nu tomato and Xiao-Nu tomatoes were pretreated with H_2_O or F8 by spraying three times with a 24-h interval between each spray, then transplanted to fields in Yuchi Township, Nantou County, or Alian Dist., Kaohsiung City. The level of disease index was recorded at 2-months post transplantation (**A**,**C**). The average of the disease index (**B**,**D**). Data represent mean ± SEM. Student’s *t*-test was used for analysis of the data in the F8-culture filtrate pretreated group and the H_2_O-pretreated control group. ** *p* < 0.01; **** *p* < 0.001.

To analyze the effect of F8-culture filtrate on tomato against WTBs, we conducted two double-blind field trials on Yu-Nu and Xiao-Nu. The first field trial was conducted in Nantou County, and the data were recorded after planting in the field for 2 months. The results showed that much milder symptoms were observed on tomato cultivar Yu-Nu pretreated with F8-culture filtrate than with H_2_O pretreatment (Figure 6A,B). 

In addition to Yu-Nu tomato, we also conducted field trials on Xiao-Nu in Alian District, Kaohsiung City. The results showed that much milder symptoms were observed on tomato cultivar Xiao-Nu pretreated with F8-culture filtrate than with H_2_O after planting in field for 2 months (Figure 6C,D). Collectively, our results indicated that F8-culture filtrate could induce effective resistance on different tomato cultivars against TYLCD in the field. 

## 4. Discussion

The application of plant immunity inducers for crop protection is an environmentally sound approach, which can reduce the use of chemical pesticides to maintain healthy crops. The data presented in this study indicates that F8-culture filtrate is an effective method to induce tomato resistance against TYLCTHV both in the greenhouse and in the field (Figure 1 and Figure 2), with no obvious decrease in tomato growth (Figure 3). In addition, we also revealed the immune pathway(s) induced by F8-culture filtrate-pretreated tomato before or after TYLCTHV infection, which provides important information for understanding how plants can effectively ward-off infection by WTBs. 

In designing the F8-culture filtrate, we have considered the cost, stability, and the method for application in the field. Instead of direct application of microbe(s) in the field, we chose F8-culture filtrate for practical application. This is to minimize environmental conditions that may affect the plant-microbe interaction and reduce the efficacy of induced resistance. F8-culture filtrate is made from inexpensive vegetable broth cultured with *Trichosporon* sp., and can be stored as a liquid at 4 °C for 2 months or as a lyophilized powder at 4 °C for at least 1 year. The powdered F8-culture filtrate can be dissolved in water before application [62]. For practical application, we only treated tomato seedlings with F8-culture filtrate before planting them in field, and no additional work was needed to induce resistance thereafter. However, profound enhanced resistance was found on F8-culture filtrate-treated tomato in the field (Figure 6). 

We previously reported that F8-polysaccharide-treated *N. benthamiana* induced a typical immune priming expression pattern in both *PR-1* and *PR-2* [62]. Intriguingly, although F8-culture filtrate induced early induction of two tomato SA-mediated immune marker genes, *PR-1* and *PR-5*, only *PR-1* showed the typical immune priming expression pattern (Figure 4A). The expression of *PR-5* was induced by F8-culture filtrate, but the induction fold was less after TYLCTHV inoculation than in the plants with F8-culture filtrate pretreatment (Figure 4B). This suggests that F8-culture filtrate can trigger the early SA-mediated immune pathway but only induce part of the SA-mediated immune priming. Alternatively, because we detected the *PR-5* expression at 4 dpi, at that time TYLCTHV may already express effector(s) to compromise part of the immune response. 

Previous reports have indicated that callose deposition has been found to confine virus infection by limiting cell-to-cell movement [79,80,81,82,83], and the SA-mediated immune pathway plays an important role in pathogen-induced callose deposition in *Arabidopsis* [81,84]. Consistently, our data indicated that F8-culture filtrate mainly induced the SA-mediated immune pathway and more callose deposits were found on tomato pretreated with F8-culture filtrate than with H_2_O (Figure 4A and Figure 5D). In addition, the gene involved in callose synthesis, *PMR4*, was up-regulated only in plants pretreated with F8-culture filtrate after TYLCTHV inoculation at 4 dpi (Figure 5F). Interestingly, the induction of callose depositions and *PMR4* was not seen in plants pretreated with F8-culture filtrate before TYLCTHV inoculation (Figure 5B). This indicated that even though we did not detect the priming of callose depositions and *PMR4* induction on F8-culture filtrate pretreated tomato before TYLCTHV inoculation, F8-culture filtrate primed the induction of tomato plants for callose depositions and *PMR4* induction.

In comparison to expression of SA-mediated immune marker genes, only certain immune marker genes from the JA or ET pathways were induced in the F8-culture filtrate pretreated group as the expression of JA responsive gene, *STH2*, and ET responsive genes, *ETR4* and *Pti4*, remained similar in all the treated groups. However, we found that a gene involved in synthesis of JA, *OPR3*, was induced at 24 hpt after F8-culture filtrate treatment and at 4 dpi after TYLCTHV inoculation on F8-culture filtrate pretreated tomato (Figure 4C). For the ethylene-mediated immune pathway, we found the gene *ETR4*, which functions as an ethylene receptor in tomato [85], is only induced in F8-culture filtrate pretreated tomato at 24 hpt (Figure 4E). This indicates inconsistent induction of JA- and ET-mediated immune marker genes. Notably, the induction of *OPR3* and *ETR4* was observed to be later than the induction of SA-mediated immune marker genes on F8-culture filtrate-treated tomato (Figure 4A,C,E). In addition, *OPR3*, but not *ETR4*, was induced after TYLCTHV inoculation (Figure 4C,E). The induction fold of *OPR3* expression was higher at 4 dpi (4.1-fold) than at 24 hpt (1.5-fold). This indicates that *OPR3* also showed a slight priming expression pattern. 

Collectively, our data indicate that F8-culture filtrate is an effective inducer of tomato defense against TYLCTHV. In addition, we found that distinct SA immunity was induced compared to our previous report on *N*. *benthamiana* [52] This indicates that F8-culture filtrate mainly induces priming of SA immune response; however, the downstream immune responses vary among different plants. Together, our findings provide important practical information for application of F8-culture filtrate for tomato resistance against TYLCTHV and also provide insights for further enhancing SA-mediated plant immunity against viruses.

## Data Availability

The data supporting the findings of this study are available within the article and Supplementary Materials.

## Supplementary Materials

The following are available online at https://www.mdpi.com/article/10.3390/v13081434/s1, Table S1: primer sequences, Figure S1: disease index of Yu-Nu tomato inoculated with tomato yellow leaf curl Thailand virus (TYLCTHV), Figure S2: symptom of Yu-Nu tomato inoculated with tomato yellow leaf curl Thailand virus (TYLCTHV). Figure S3: fresh weight of Yu-Nu tomato with H_2_O or F8-culture filtrate pretreatment.
