# Supplementary material for: Fungal F8-Culture Filtrate Induces Tomato Resistance against Tomato Yellow Leaf Curl Thailand Virus
Source: Viruses. 2021 Jul 23;13(8):1434. doi: 10.3390/v13081434 (PMC8402638; doi:10.3390/v13081434)
Supplement: Supplementary file 1 [file viruses-13-01434-s001.zip › viruses-1276240-supplementary.pdf]

Supplementary Materials

**Table S1. Primers sequence**			
**Genes**	**Accession no.**	**Forward**	**Reverse**
SlDCL2	*Solyc 06g048960*	GAGTGCCATAATGCACGAGG	TTGCCAATAACTATCTGCTGTG
SlDCL4	*Solyc 07g005030*	CTTTGTTGAACTACCTCCTG	ATTGCTAACTCCCTCCC
SlAGO1a	*Solyc 06g072300*	CTATCAGCCCCCAGTTACGTTTG	ATCACCCTTTTGACATTCTCCTTG
SlPR1	NM_001247429	AACGCTCACAATGCAGCTCGT	AAGGTCCACCAGAGTGTTGC
SlPR5	NM_001247422	GCAACAACTGTCCATACACC	AGACTCCACCACAATCACC
SlPti4	NM_001347076	TCGTCGGGAAACGGTTCCAT	GACATCCAACTTGCATGACACTTG
SlETR4	NM_001247276	GGTAATCCCAAATCCAGAAGGTTT	CAATTGATGGCCGCAGTTG
SlOPR3	NM_001246944	ATGTTGGTCGTGCATCTCAT	GGTTCCAATTGCTCTTGGTT
SlSTH2	*Solyc05g054380*	GAAGGGGATCCATTGGGACAA	TTCCCATAGCACTATCTTTTCC
SlPMR4	*Solyc07g053980*	GCCGGCGGCGAGACAAGTTT	CAGCGCCAGCCAGTCAAGCA

TYLCTHV DNA-A	GU723754	TATTCGGTGTTGCACCTTGA	AGGCGGAATTCCCACTATCT
TYLCTHV DNA-B	GU723754	GGGTCTTTGGGGGAGAAA	TCGGATAGGACTGAATTTCAG
TYLCTHV DNA-A_Semi	GU723754	TTTCCGTACTTTGTGTTTGATTGCCAGT	CATACAACGGAAACCGTGGACG
Slβ-Actin	TC178617	GGAAAAGCTTGCCTATGTGG	CCTGCAGCTTCCATACCAAT

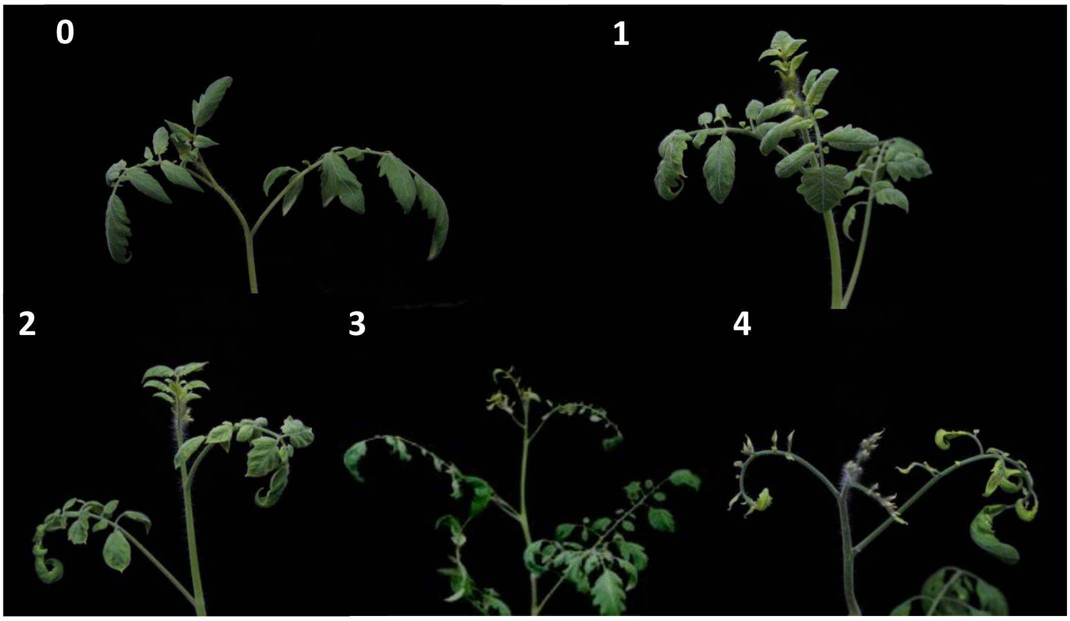

Figure S1. Disease index of Yu-Nu tomato inoculated with tomato yellow leaf curl Thailand virus (TYLCTHV). Yu-Nu were inoculated with TYLCTHV by white fly vector. The disease index was classified into 5 levels according to the degree of yellowing, stunting, and wilting at 28 dpi to 35 dpi. 0 = no visible symptoms, inoculated plants show the same growth and development as non-inoculated plants; 1 = slight yellowing and minor curling of leaflet ends; 2 = wide range of leaf yellowing, curling and cupping, with some reduction in size; 3 = wide range of leaf yellowing, pronounced leaf cupping and curling; 4 = very severe plant stunting and leaf yellowing, pronounced leaf cupping and curling, and plant ceased to grow.

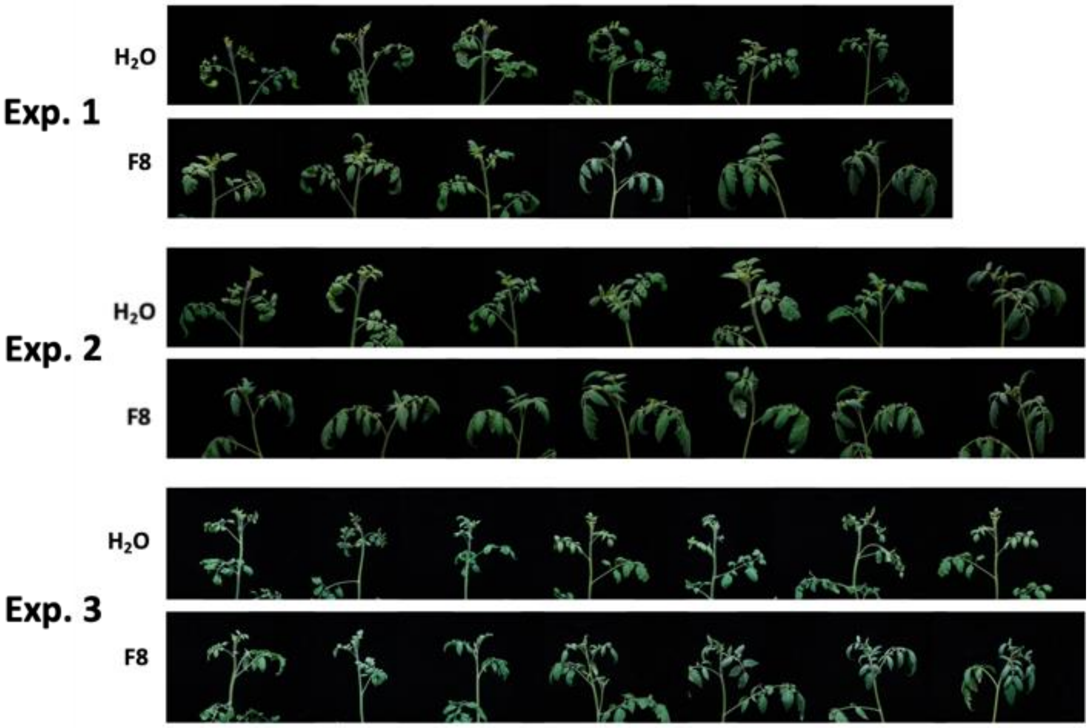

Figure S2. Symptom of Yu-Nu tomato inoculated with tomato yellow leaf curl Thailand virus (TYLCTHV). Disease symptoms of tomato plants pretreated with H_2O or F8-culture filtrate inoculated with TYLCTHV in 3 independent replicates of experiments (Exp.1, Exp. 2, and Exp. 3). The photos were taken at 28 dpi.

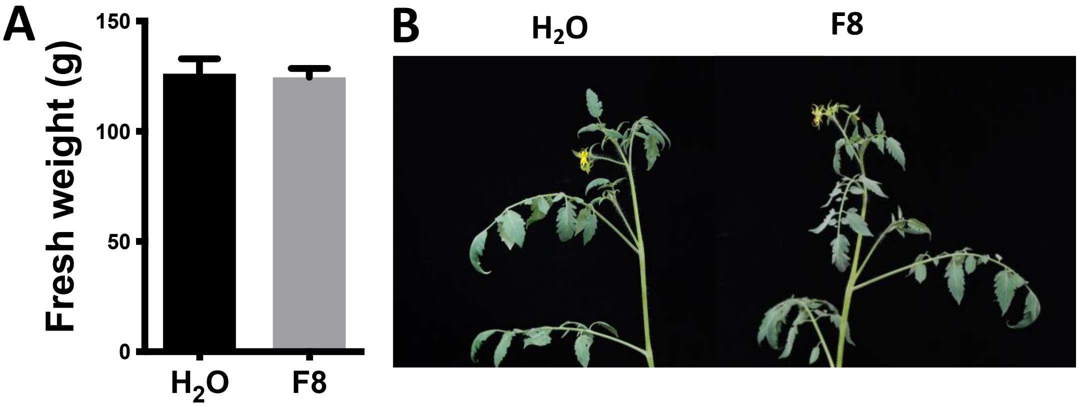

Figure S3. Fresh weight of Yu-Nu tomato with H_2O or F8-culture filtrate pretreatment of repeated experiment set. **(A)** The average fresh weight was recorded for each treatment group (*n* = 8). **(B)** The phenotype of H_2O or F8-culture filtrate at 5 weeks post treatment.
